# Lenvatinib as an urgent initial therapy in thyroid cancer with remarkable laryngotracheal invasion

**DOI:** 10.1530/ETJ-25-0212

**Published:** 2025-10-08

**Authors:** Hiroshi Katoh, Riku Okamoto, Yuka Ozawa, Takaaki Tokito, Mariko Kikuchi, Takafumi Sangai

**Affiliations:** Department of Breast and Thyroid Surgery, Kitasato University Hospital, Sagamihara, Kanagawa, Japan

**Keywords:** tracheal invasion, laryngeal invasion, lenvatinib, turn-around-time, thyroid cancer

## Abstract

**Objectives:**

In thyroid cancer with marked laryngotracheal invasion, life-threatening airway stenosis necessitates urgent procedures such as extensive curative surgery, tracheostomy, stenting, or laser bronchoscopy. These interventions are invasive and may significantly compromise quality of life. In anaplastic thyroid cancer (ATC), the delay during genetic testing turnaround time before initiating targeted therapy poses an additional therapeutic challenge. This study aimed to assess lenvatinib (LEN) as an initial and bridging treatment to rapidly alleviate airway stenosis and avoid emergency invasive interventions.

**Methods:**

This retrospective study analyzed 14 patients with remarkable laryngotracheal invasion among 69 thyroid cancer patients treated with multikinase inhibitor(s). All 14 patients received LEN as first-line or post-paclitaxel treatment. Response was assessed by CT imaging per RECIST 1.1, with particular attention to changes in tumor size and airway diameter. Symptom improvement and adverse events, such as fistula formation, were also recorded.

**Results:**

Of the 14 patients, 13 showed tumor reduction and airway improvement on initial CT post-LEN induction. Median response rate was 28.4%, with airway diameter improving by 15.9% on the initial CT. Airway symptoms resolved in a median of 3 days. One patient developed a tumor-tracheal fistula, managed with LEN dose adjustment. LEN was also successfully used as a bridging therapy before BRAF-targeted treatment in ATC cases.

**Conclusions:**

Initial LEN therapy rapidly alleviates airway stenosis in advanced thyroid cancer with laryngotracheal invasion, offering a non-invasive alternative to emergency procedures under careful monitoring for fistula formation. LEN is especially valuable as a bridging therapy during the genetic testing period in ATC.

## Introduction

Locally advanced thyroid cancer involves adjacent vital structures such as the trachea, larynx, common carotid artery, and esophagus (1). Remarkable laryngotracheal invasion can cause severe airway stenosis, requiring urgent treatment including radical resection, tracheostomy, and so forth. Undoubtedly, the initial treatment of locally advanced differentiated thyroid cancer (DTC) is radical resection; however, this may require sacrificing the critical structures in order to achieve complete curative resection. Therefore, such aggressive and extended resection raises a dilemma between curability and impairment of quality of life (QoL) or risk of postoperative severe complications. Moreover, urgent treatment is necessary for severe airway stenosis caused by laryngotracheal tumor invasion. In such life-threatening cases with laryngotracheal invasion of thyroid cancer, urgent curative surgery combined with tracheal resection or laryngectomy, tracheostomy, palliative stenting, or laser bronchoscopy would be treatment options (2, 3, 4, 5, 6, 7, 8). However, the risks of significant morbidity and mortality raise questions about conducting aggressive surgery, and tracheostomy decreases QoL. Tracheostomy is sometimes difficult and even life-threatening in cases of large anaplastic thyroid cancer (ATC) that extends from the larynx to the mediastinum (9, 10, 11, 12, 13). Palliative stenting is often difficult for tumors located close to the vocal cords (14, 15). Laser bronchoscopy and palliative stenting are usually invasive for symptomatic severe tracheal stenosis, especially in elderly patients. Recently, conversion surgery after multikinase inhibitor (MKI) treatment has been reported for locally advanced thyroid cancer (16, 17, 18, 19, 20, 21, 22, 23, 24, 25, 26, 27). Moreover, in ATC, since remarkable efficacy of BRAF inhibitors has been reported (28, 29), dabrafenib/trametinib treatment for *BRAF^V600E^*-variant ATC is recommended as an initial treatment even in resectable disease in the NCCN guidelines 2025 (https://www.nccn.org/professionals/physician_gls/pdf/thyroid.pdf). This neoadjuvant use of molecular targeting drugs may provide potential curability in unresectable disease and increase the possibility of functional preservation surgery. On the other hand, particularly in ATC, even if a core needle biopsy (CNB) is submitted for genetic testing simultaneously with pathological diagnosis without delay, the turn-around-time (TAT) of genetic testing usually takes 7–14 days in both NGS-based assays (30, 31) and PCR-rSSO *BRAF^V600E^* tests (JSR Life Science and SRL Japan, https://test-directory.srl.info/akiruno/test/detail/00U593700); initiation of treatment is therefore delayed until after this period. Accordingly, effective bridging therapy during TAT is urgently needed for patients with severe laryngotracheal stenosis. Here, we report the efficacy of lenvatinib (LEN) as an initial treatment in locally advanced DTC and ATC with remarkable laryngotracheal invasion, including symptomatic airway stenosis.

## Patients and methods

**Figure 1:**
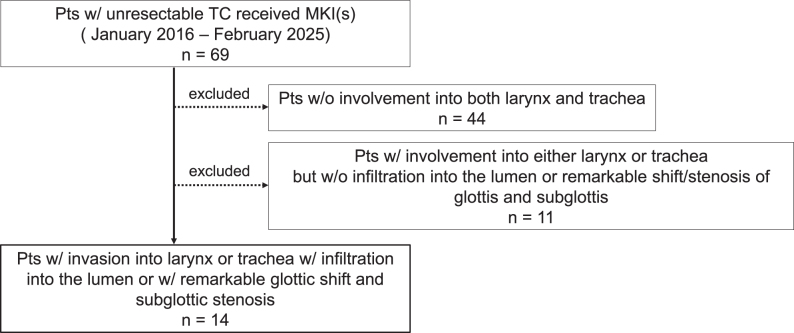
Among 69 patients who were treated with MKI(s), 44 cases without involvement of either larynx or trachea were excluded. Eleven cases with suspicious involvement into either larynx or trachea but without infiltration into the lumen or remarkable shift/stenosis of glottis and subglottis were further excluded from the study. The remaining 14 patients had obvious invasion into larynx or trachea, with infiltration into the tracheal lumen or with remarkable glottic shift and subglottic stenosis.

**Figure 2:**
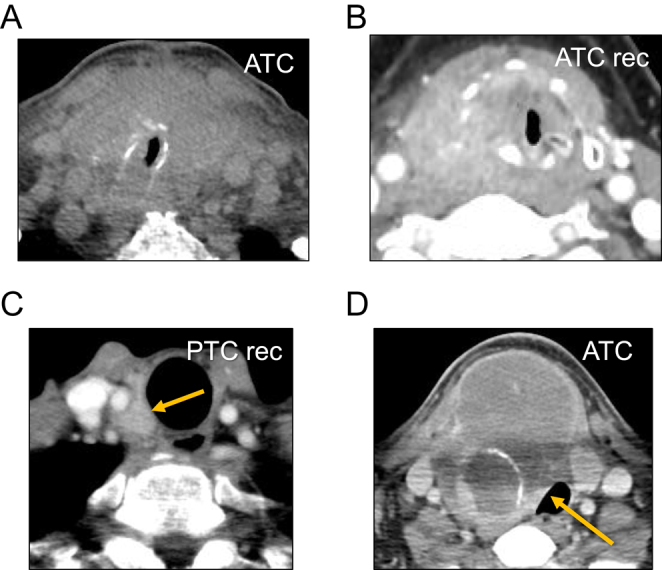
Typical CT scans images of airway involvement. (A and B) Remarkable invasion into trachea (A) or larynx (B) with remarkable shift and stenosis. (C and D) Typical cases of tumors that were widely attached to trachea without infiltration into the lumen, with preserved border of the tracheal wall. These cases were excluded from the present study, suspicious of tracheal involvement; rec, recurrence.

We retrospectively evaluated 69 patients who were treated with MKIs (LEN, sorafenib, or vandetanib) for unresectable thyroid cancer under insurance coverage of the Japanese healthcare system at Kitasato University Hospital between January 2016 and March 2025. All cases were primarily managed by surgeons throughout the clinical course. Final treatment decisions were made through multidisciplinary discussions, including medical oncologists, with surgeons taking the lead. Of these patients, 25 were diagnosed with involvement of either the larynx or trachea on CT scan ± MRI as well as laryngotracheal fiberscopy. Laryngeal fiberscopy with tracheal assessment was routinely performed to evaluate evident laryngeal or intratracheal invasion as well as vocal cord paralysis. In cases with severe laryngeal stenosis or edema due to laryngeal involvement (cases 6, 8, 13, and 14), intratracheal examination was avoided. In cases without airway compromise, such as stridor or dyspnea, and when the clinical course allowed sufficient time, MRI was performed to evaluate tissue boundaries. When esophageal invasion was suspected, an additional upper gastrointestinal endoscopy was conducted to objectively assess luminal involvement. This approach ensured thorough assessment while prioritizing patient safety. Among them, 14 patients with remarkable invasion into either the trachea or/and larynx were assessed in this study (Fig. 1). Remarkable laryngotracheal invasion was defined as obvious invasion into the tracheal lumen or obvious infiltration into the larynx with a remarkable shift of the glottis and subglottic stenosis on CT scan (Fig. 2A and B). Patients suspicious of tracheal involvement without invasion into the tracheal lumen or infiltration into the larynx without glottic shift and subglottic stenosis were excluded from the present study. As typically shown in Fig. 2C and D, cases widely attached to the trachea without infiltration into the lumen with preserved borders of the tracheal wall were excluded from this study. Six patients had symptomatic airway stenosis with stridor (six patients) ± dyspnea (one patient). For these patients, the time to disappearance or improvement of symptoms was assessed. Pathological diagnosis was initially performed by CNB, except in cases 3 and 8. These 2 cases with recurrent poorly differentiated thyroid cancer (PDTC) (case 8) and recurrent medullary thyroid cancer (MTC) (case 2) were diagnosed with fine-needle aspiration cytology referring to the first surgical specimen of the primary tumor. In recent three ATC cases (cases 12, 13, and 14), companion genetic tests (MEBGEN^TM^ BRAF3 kit and Oncomine Dx TT^TM^) were submitted for simultaneous testing to initiate treatment promptly. In cases 9, 10, and 12, which proceeded to conversion surgery, pathological diagnoses were further confirmed by surgical specimens. Pathological diagnoses of either CNB or surgical specimens were rendered independently by three board-certified pathologists with expertise in thyroid pathology; for ATC and PDTC cases, the final diagnosis was further confirmed through a formal review by the pathology department of our institution. Case 5, in which a complete response (CR) was achieved with LEN for recurrent ATC, was enrolled in a multicenter collaborative phase II study of LEN (HOPE study) (32), and the diagnosis was additionally confirmed by an independent central pathological review. The disease was staged according to the medical records and the 8th edition of the UICC TNM staging system. Radiological diagnosis was confirmed by institutional radiologists.

Although the efficacy of LEN in combination with pembrolizumab for advanced thyroid cancers, including ATC, has been reported (26, 33, 34, 35, 36), the combination therapy is not yet approved for thyroid cancer in Japan. According to the 2024 revised clinical guidelines on the management of thyroid tumors by the Japan Association of Endocrine Surgery (37), LEN monotherapy is approved as a treatment option for ATC in the absence of druggable gene alterations. Therefore, LEN monotherapy was used in the present cohort. Response to MKI treatment in tumor size was evaluated on CT scan according to the Response Evaluation Criteria in Solid Tumors version 1.1 (RECIST 1.1) criteria (38). In this study, objective response in tumor size was evaluated in 3 dimensions (the greatest and perpendicular diameters in the axial plane image, and the longitudinal diameter in the coronal plane image) on CT scan. The response in tracheal or laryngeal invasion was evaluated by measuring the tracheal or subglottic diameter in the axial plane image on CT scan. Following initiation of LEN, the first CT follow-up was performed, barring exceptional circumstances, within 2 weeks – more recently within 1 week – and subsequently at 2-week intervals until week 8, in order to enable early detection of severe adverse events (AEs) such as tumor–tracheal fistula. AEs were defined as any unfavorable and unintended sign(s), symptom(s), or disease(s) (new or worsening) associated with MKI treatment and were graded according to the Common Terminology Criteria for Adverse Events (CTCAE) version 5.0.

This study was approved by the Institutional Review Board (IRB) of the Kitasato University School of Medicine (approval no. B23-057) and conformed to the clinical research guidelines of the IRB of the Kitasato University School of Medicine and the Declaration of Helsinki (as revised in 2013). All individuals provided written informed consent for pathologic assessment of their samples, routine laboratory tests, and analysis of their clinical data. The approach of opt-out consent was employed for this retrospective analysis.

## Results

### Patient and disease characteristics

Table 1 summarizes the demographics of 14 patients (ten primary tumors and four recurrent diseases) with remarkable invasion into the larynx or trachea. The patients were histologically comprised of ten ATCs, two poorly differentiated thyroid cancers (PDTCs), one papillary thyroid cancer (PTC), and one MTC. The two PDTCs were derived from PTC and follicular thyroid cancer, respectively. Invasion into the trachea, larynx, or both was observed in eight (57%), four (29%), or two (14%), respectively. Of them, six patients (five ATCs and one PTC) showed stridor, and one patient with ATC further complained of severe dyspnea and underwent emergent tracheostomy. All of them were treated with LEN. The median observation period from LEN initiation was 386 (95–2,453) days, and the median age at LEN initiation was 75 (52–82) years, with nine females and five males. Four patients with ATC were initially treated with weekly paclitaxel (w-PTX) at a dose of 80 mg/m^2^. Clinical response was not observed in these four patients, leading to consequent LEN treatment, as in other patients who were directly initiated with LEN. No patient was treated with external beam radiation therapy before initiating LEN. LEN was initiated at a dose of either 24 or 14 mg/d. In recent practice (recent four cases), to mitigate the risk of serious AEs (particularly fistula formation), we implemented a protocol whereby treatment is initiated at a reduced dose of 14 mg in combination with a weekend-off regimen (ATC: 1-day drug holiday; DTC: 2-day drug holiday). Before adopting this protocol, patients either commenced treatment at 14 mg or initiated therapy at 24 mg with a planned dose reduction within the first week. Since BRAF/MEK inhibitors and their companion diagnostic test (MEBGENTM BRAF3 kit) were approved for insurance coverage in November 2023 in Japan, patients treated before case 10 received long-term LEN therapy. Moreover, cases before case 8 were managed in an era when clinical trials involving BRAF inhibitors had not yet been initiated. Although both case 6 and case 10 were found to harbor the *BRAF V600E* mutation by comprehensive genomic profiling (CGP) (FoundationOne CDx) during the period when clinical trials of BRAF-targeted therapy were ongoing in Japan, they were deemed ineligible for trial enrollment due to the extent of tumor invasion and thus could not receive BRAF inhibitors.

**Table 1:** List of patients with remarkable invasion into larynx or trachea. (The cases are listed in chronological order from top to bottom).

Case	Gender	Age	HIST	Tumor	Airway invasion	Obs sym	Prior CTx	Initial dose of LEN (mg)	Day of first CT*	Response on first CT*			Days to change^⁑^	TAF
										ORR (%)	RECIST criteria	Improve-ment^‡^		
1	F	74	ATC	Primary	Trachea	No	w-PTX	24	14	28.4	SD	9.2	NA	No
2	F	71	ATC	Primary	Trachea	Yes	w-PTX	14	14	55.9	PR	60.9	NA^#^	Yes
3	M	52	MTC^¶^	Recurrence	Trachea	No	No	14	18	67.3	PR	5.8	NA	No
4	F	71	ATC	Primary	Trachea	No	w-PTX	24	18	−48.9^†^	PD	−2.9^†^	NA	No
5	F	75	ATC	Recurrence	Trachea	No	No	24	14	16.3	SD	5.4	NA	No
6	F	66	ATC	Recurrence	Larynx	Yes	No	24	6	15.3	SD	27.5	3/7	No
7	M	81	ATC	Primary	Trachea	Yes	No	24	7	54.6	PR	37.1	2/5	No
8	M	81	PDTC	Recurrence	Both**	No	No	14	30	29.2	SD	15.9	NA	No
9	F	77	PDTC	Primary	Larynx	No	No	24	21	47.2	PR	16.7	NA	No
10	M	70	ATC	Primary	Larynx	No	No	24	14	29.9	SD	12.2	NA	No
11	F	82	PTC	Primary	Trachea	Yes	No	14 (5 days/week)	14	14.6	SD	39.3	3/5	No
12	F	81	ATC	Primary	Trachea	Yes	No	14 (6 days/week)	5	33.5	PR	17.0	3/11	No
13	M	80	ATC	Primary	Larynx	No	w-PTX	14 (6 days/week)	5	27.1	SD	22.2	NA	No
14	F	80	ATC	Primary	Both**	Yes	No	14 (6 days/week)	7	12.0	SD	5.0	3/3	No

* First CT scan after LEN initiation.

** Both indicates invasion into both larynx and trachea.

^‡^ Improvement of airway diameter on first CT* (%).

^⁑^ Days to improvement/disappearance of symptoms.

^#^ Emergent tracheostomy was performed in this patient.

^†^ Response to treatment was not observed, and disease progressed in this case.

^¶^ In Japan, LEN was approved and reimbursed for unresectable thyroid cancer, including MTC, in May 2015, whereas vandetanib (VAN) was approved and reimbursed later, in late December 2015. Consequently, this patient received LEN as first-line therapy and VAN as second-line therapy (Table 2). Cabozantinib is not reimbursed in Japan.

CTx, chemotherapy; LEN, lenvatinib; ORR, objective response rate; F, female; M, male; ATC, anaplastic thyroid cancer; HIST, histology; MTC, medullary thyroid cancer; PDTC, poorly differentiated thyroid cancer; PTC, papillary thyroid cancer; w-PTX, weekly paclitaxel; NA, not applicable; Obs sym; obstructive symptom; TAF, tumor-airway fistula.

### LEN treatment rapidly improved airway stenosis-related symptoms

**Figure 3:**
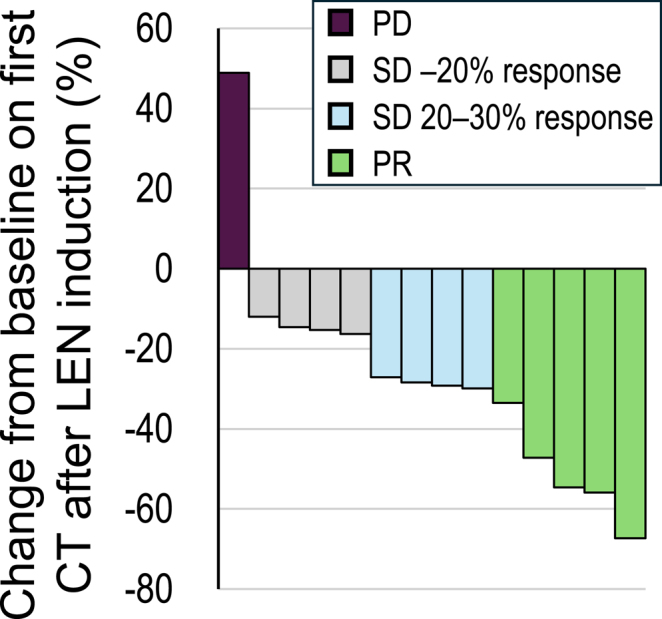
Waterfall plot of percentage change of the primary tumor diameters on the first CT scan after lenvatinib (LEN) initiation.

**Figure 4:**
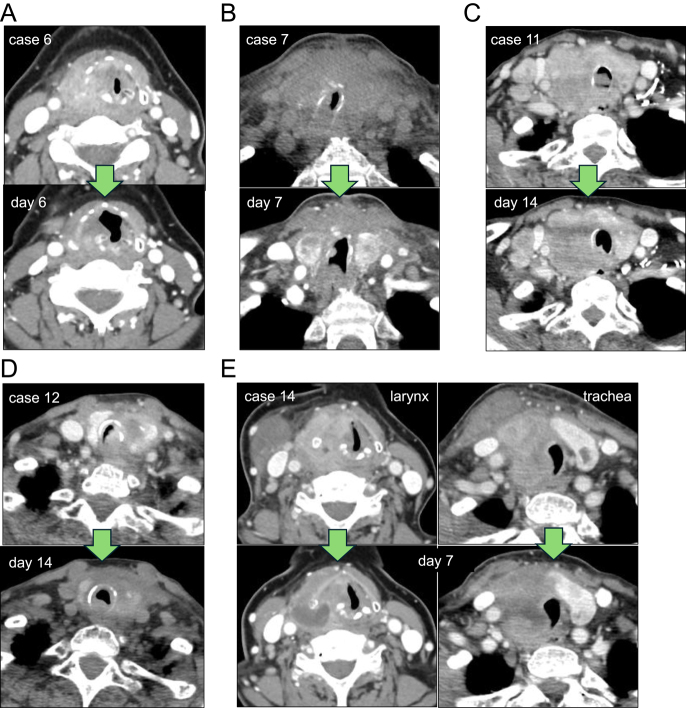
Efficacy of LEN in patients with stridor caused by tumor invasion on the first CT after LEN initiation. Four patients (cases 6, 7, 12, and 14) with ATC and one patient (case 11) with PTC. Airway stenosis was immediately and remarkably improved after LEN initiation in cases 6, 7, 11, and 12 (A, B, C, D), while minor response on airway stenosis was observed in case 14 (E).

As shown in Table 1 and Fig. 3, LEN efficacy, including partial response (PR) and stable disease (SD) with minor response, was observed in 13 out of 14 patients (92.9%) on the first CT after LEN initiation. The median objective response rate (ORR) was 28.4% (mean 27.3%) on the first CT. Similarly, the actual airway diameter was improved in 13 patients, except for one ATC case, with the median improvement of airway diameter at 15.9% (mean 19.4%). Correspondingly, stridor was immediately improved in all patients (except one ATC patient who underwent tracheostomy). Of note, the mean time to improvement and disappearance of airway obstructive symptoms was rapid, with 2.8 days (median 3 days) and 6.2 days (median 5 days), respectively. The images of CT scan of these five patients are shown in Fig. 4. Importantly, airway obstructive symptoms were immediately improved even in patient (case 14), with minor improvement of airway stenosis (Table 1, Fig. 4E).

**Figure 5:**
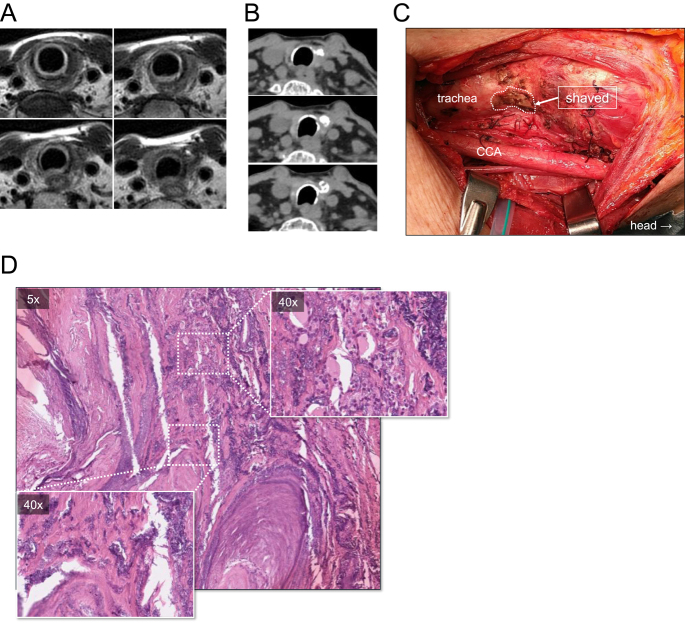
Case 12 ATC patient with airway obstructive symptoms successfully treated with bridging LEN treatment and subsequent DT, leading to conversion surgery. (A and B) Neck MRI and CT scan images after 56 days of LEN treatment and subsequent DT treatment (6 (MRI) and 8 (CT) months) indicate dramatic response to treatment, and the area of tracheal invasion was no longer detectable. (C) Intraoperative image shows shaved trachea for tumor involvement. Intraoperative frozen section diagnosis of the invasive front in the tracheal wall revealed non-viable atypical cells. Dotted line indicates shaved area. (D) Final pathological diagnosis was CR with nodular changes consisting of fibrosis, hyalinization, coarse calcification deposits, and foamy cells. Regenerative follicles in the surrounding area and some roundly arranged epithelial cells without atypia were observed.

For patients other than those with ATC, LEN was initiated with the expectation that tumor shrinkage might enable conversion surgery. Case 9 subsequently underwent conversion surgery, and case 11 is scheduled for conversion surgery (Table 2). In ATC cases without druggable gene alterations, conversion surgery was attempted without delay when sufficient tumor shrinkage was achieved with LEN. Case 10 proceeded directly to conversion surgery after LEN treatment, as BRAF-targeted therapy was not yet reimbursed in Japan at that time. The average best ORR of LEN was 55.0% (median 65.8%) (Table 2). Case 5 with ATC recurrence showed CR with LEN treatment, and there has been no sign of recurrence 54 months after LEN withdrawal. LEN treatment failed in six patients (four ATC, one PDTC and one MTC) over an average of 765.5 days (56–1,021 days). In the recent three cases with ATC (cases 12, 13, and 14), dabrafenib/trametinib (DT) therapy was conducted after bridging LEN treatment during the TAT of genetic testing. In case 12, with airway obstructive symptoms (stridor), after remarkable improvement of tracheal obstruction by LEN treatment with 65.8% PR (Fig. 4D, Tables 1 and 2), subsequent DT therapy exhibited further tumor shrinkage to 90.3% PR (Fig. 5A and B). On neck MRI, the area of tracheal invasion was no longer identifiable (Fig. 5A). In fact, curative resection was performed with tracheal wall shaving without tracheal resection (Fig. 5C). Intraoperative frozen section diagnosis of the invasive front in the shaved tracheal wall revealed non-viable atypical cells, and the final pathological diagnosis was CR (Fig. 5D). The excellent treatment course in case 12 could not have been observed without the bridging LEN administration to manage the critical and severe airway stenosis caused by tumor invasion.

**Table 2:** Therapeutic course after LEN initiation in patients w/remarkable invasion into larynx or trachea. The cases are listed in chronological order in correspondence with Table 1.

Case	Gender	Age	HIST	Best response of LEN		LEN period (days)	Actinonable genetic variant	Next line	CONV surgery	TTF of LEN, days	OS after LEN start, days	Survival status^#^
				ORR (%)	RECIST criteria							
1	F	74	ATC	68.1	PR	326	NA	None	No	287	423	Deceased
2	F	71	ATC	76.3	PR	108	NA	None	No	NA^¶^	151	Deceased
3	M	52	MTC	67.3	PR	407	Somatic *RET*	VAN	No	388	1,347	Deceased
4	F	71	ATC	−48.9^†^	PD	89	NA	None	No	56	99	Deceased
5	F	75	ATC	100	CR	858	NA	– ^##^	No	NA	2,565	Alive
6	F	66	ATC	51	PR	1,089	* *BRAF V600E, TERT, PIK3CA*	None	No	1,021	1,314	Deceased
7	M	81	ATC	75.6	PR	57	NA	None	No	NA^¶^	144	Deceased
8	M	81	PDTC	29.2	SD	622	NA	None	No	510	722	Deceased
9	F	77	PDTC	85.5	PR	1,211	* *NRAS, KRAS*	On going	Yes	NA	1,343	Alive
10	M	70	ATC	44.2	PR	383	* *BRAF V600E, TERT, PIK3CA, TP53*	None	Yes	315	386	Deceased
11	F	82	PTC	87.8	PR	598	NA	On going	No^‡^	NA	730	Alive
12	F	81	ATC	65.8	PR	50	** *BRAF V600E*	DT	Yes	NA	484	Alive
13	M	80	ATC	27.2	SD	12	** *BRAF V600E*	DT	No	NA	95	Deceased
14	F	80	ATC	40.9	PR	20	** *BRAF V600E*	DT	No^‡^	NA	253	Alive

* Assessed by FoundationOne CDx.

** Assessed by either MEBGEN3 BRAF kit or/and Oncomine Dx target test.

^#^ Survival at the final follow up.

^##^ Discontinued due to complete response.

^¶^ LEN treatment was withdrawn according to the patients’ wishes.

^†^ Response to treatment was not observed and disease progressed in this case.

^‡^ Conversion surgery is planned in these patients.

TTF, time to failure; OS, overall survival; NA, not assessed; VAN, vandetanib; DT, dabrafenib/trametinib; HIST, histology; CONV, conversion.

### Tumor-tracheal fistula caused by LEN-induced tumor shrinkage

**Figure 6:**
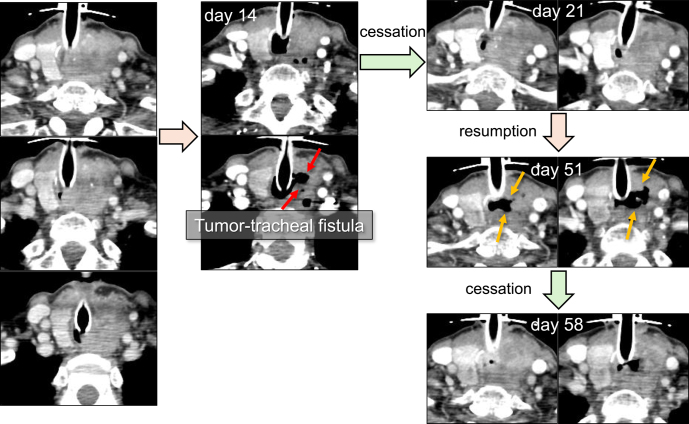
Tumor-tracheal fistula caused by LEN treatment in a patient with ATC (case 2). Tumor-tracheal fistula was first observed 14 days after LEN initiation on CT scan. Since tumor-tracheal fistula was restored by tumor regrowth after 7 days of LEN treatment cessation, LEN treatment was resumed. After resumption of LEN treatment, tumor-tracheal fistula relapsed, and the patient required re-cessation of LEN treatment.

The most critical adverse effect of LEN is the development of fistula or perforation of adjacent critical structures (39, 40). In this study, tumor-tracheal fistula was observed in one patient with ATC (case 2). The patient underwent emergent tracheostomy for severe tracheal obstruction caused by tumor invasion (Table 1, Fig. 6 left). After failure of prior w-PTX at a dose of 80 mg/m^2^, LEN treatment was started at 14 mg daily. Tumor-tracheal fistula was first observed 14 days after LEN initiation on CT scan (Fig. 6 middle), and LEN administration was immediately stopped. Since tumor-tracheal fistula was restored by rapid tumor regrowth after 7 days of cessation of LEN treatment (Fig. 6 right upper), LEN treatment was resumed at a reduced dose of 10 mg. Tumor-tracheal fistula recurred after 30 days of LEN readministration (Fig. 6 right middle). After 7 days of LEN cessation, tumor-tracheal fistula was restored by tumor regrowth (Fig. 6 right bottom), and LEN treatment was restarted at 8 mg daily. Since then, there has been no recurrence of the tumor-tracheal fistula, and the tumor has remained stable. Although genetic testing was not available during the period, tumor-tracheal fistula and tumor progression were controlled by LEN treatment with periodical cessation. According to the patient’s wishes for best supportive care, LEN was withdrawn 4 months after LEN initiation. Six weeks after withdrawal of LEN, the patient died of respiratory failure resulting from tumor progression.

## Discussion

This study evaluated the clinical usefulness of LEN treatment in thyroid cancer with remarkable airway invasion. Most patients (13 out of 14 patients) showed an objective response (PR and SD with minor response) at the first CT scan after LEN initiation. Importantly, all five patients with stridor exhibited immediate improvement of airway obstructive symptoms within 3 days of LEN treatment, suggesting that LEN can be an initial treatment option as a non-traumatic therapy. These results provide clinical impact, particularly for ATC patients with severe airway stenosis caused by tumor invasion. In these patients, LEN therapy is remarkably effective as a bridging therapy during TAT of genetic testing.

According to the excellent efficacy in the SELECT study (41), LEN has been accepted as the preferred regimen in RAI-refractory DTC in the NCCN guidelines 2025 (https://www.nccn.org/professionals/physician_gls/pdf/thyroid.pdf). Moreover, conversion surgery after LEN treatment has been reported for locally advanced thyroid cancer (16, 17, 18, 19, 20, 21, 22, 23, 24, 25, 27). However, the phase II study of ATC conducted in Europe showed poor outcomes with LEN and was terminated early due to lack of efficacy, with a response rate of approximately 3% (42). In the Japanese phase II study of ATC (HOPE study), the 1-year overall survival (OS) was 11.9%, which was higher than that reported in Europe, though no clear improvement in OS was demonstrated (32). Accordingly, LEN monotherapy for ATC patients is excluded from NCCN guidelines. Nevertheless, in the HOPE study, durable tumor regression, including CR, was achieved in selected cases, and more than half of the patients experienced at least temporary tumor shrinkage, supporting the rationale for LEN as a bridging treatment option. Other previous literature has also reported that LEN may be beneficial for the long-term prognosis of selected patients with unresectable ATC (43, 44). Taken together with the present results, initial LEN therapy would be a promising treatment option for ATC with remarkable airway invasion, at least as a bridging therapy during TAT. In addition, three DTC patients with airway invasion rapidly responded to LEN therapy. Among them, LEN therapy immediately improved stridor in a PTC patient (case 11) and resulted in 87.8% best objective response (Tables 1 and 2).

Despite the prompt antitumor efficacy of LEN, the occurrence of tumor-tracheal fistula in patients with tracheal invasion represents a serious and potentially fatal AE (39, 40). In this study, one patient (case 2) developed LEN-induced tumor-tracheal fistula, in whom early detection of tumor-tracheal fistula via CT, combined with timely interruption of LEN and its subsequent reintroduction at a reduced dose, enabled temporary control of both tumor and fistula progression (Fig. 6). LEN-induced remarkable tumor shrinkage (≥10% tumor size decrease) occurs rapidly within the first 8 weeks from LEN initiation, followed by a gradual decrease in the relatively late phase (39, 45, 46). In fact, tumor-tracheal fistula in case 2 was detected 14 days after LEN induction. Accordingly, careful observation by frequent CT scans (e.g. every 2 weeks) would be necessary for early detection of tumor-tracheal fistula, particularly during the first 2 months from LEN initiation.

Previous report suggested that the starting dose was not relevant for the development of fistula (40). On the other hand, tumor-shrinkage rate has been reported to be dose-dependent (45, 46, 47), and a rapid tumor-shrinkage rate may be a risk for fistula development (46). Planned drug holidays of LEN have been reported to contribute to decreased intolerable AEs, leading to favorable long-term prognosis in DTC (48, 49). In addition, tumor-suppressive effects of LEN could be expected even with a low dose (44). Collectively, considering that tumor control can be achieved even with low-dose LEN, dose reduction at initiation and/or weekend(s)-off (planned drug holidays) schedules may be considered as treatment options for cases with marked tracheal invasion.

The advantages of initial LEN treatment in thyroid cancer with remarkable tracheal invasion are that initial LEN therapy can be initiated promptly, provides rapid improvement of airway stenosis-associated symptoms, and helps avoid urgent invasive and traumatic procedures (e.g. tracheostomy, palliative stenting, or laser bronchoscopy), which is also beneficial from a QoL perspective. Notably, in patients with ATC, LEN can serve as a bridging therapy during the TAT of genetic testing. Particularly in the absence of actionable genetic alterations, the treatment-free interval during TAT may represent a missed therapeutic opportunity for patients. Careful clinical monitoring with CT scans is, of course, essential for the early detection of fistula formation or other risks for life-threating AEs, such as lethal hemorrhage.

In the management of ATC, simultaneous submission of a CNB from a large neck mass for both histopathological and molecular assays is crucial to facilitate early diagnosis (50, 51). Recently, the clinical utility of BRAF immunohistochemistry (IHC) has been reported (52, 53, 54, 55). In Japan, although BRAF IHC is actually performed in certain institutions at their own expense, eligibility for insurance coverage of BRAF inhibitors requires confirmation of the mutation through an approved genetic companion diagnostic test. This prerequisite may result in prolonged TAT. BRAF IHC may have the potential to provide advantages in terms of both TAT and cost-effectiveness.

It is generally acknowledged that surgical intervention, including radical resection or urgent invasive and traumatic procedures (e.g. tracheostomy, palliative stenting, or laser bronchoscopy), constitutes the first treatment modality in DTC cases other than ATC. Nevertheless, in select cases – such as case 11, involving an elderly patient presenting with stridor due to tracheal invasion – provided a comprehensive explanation of the associated risks is given and informed consent is duly obtained, non-invasive LEN treatment may represent a viable therapeutic alternative. This is particularly pertinent when such an approach offers the potential for prompt alleviation of airway obstruction symptoms. Furthermore, in certain patients, LEN administration may contribute to tumor volume reduction, thereby potentially obviating the need for extensive surgical procedures (16, 17, 18, 19, 20, 21, 22, 23, 24, 25, 27).

This study has several limitations. First, it was retrospectively conducted in a single institution with a small sample size, which may reduce the generalizability of the findings. In addition, the absence of a control group hinders the objective evaluation of therapeutic efficacy. While multicenter studies will be necessary to validate and expand upon these findings, the rapid progression and need for urgent clinical management in ATC involving the airway pose substantial challenges to conducting large-scale multicenter studies within a short timeframe. In light of the pressing need to support physicians managing patients with similarly aggressive disease, we believe that the findings – while preliminary – may offer clinically meaningful insights and could serve as a useful contribution to the existing literature.

In conclusion, initial LEN therapy exhibited rapid improvement of airway obstructive symptoms in patients with marked laryngotracheal invasion, allowing avoidance of urgent invasive and traumatic procedures. Bridging LEN therapy would provide significant impact, particularly for ATC patients with severe airway stenosis caused by tumor invasion during TAT of genetic testing. Therefore, initial LEN therapy should be considered for patients with remarkable laryngotracheal invasion under careful monitoring for fistula formation.

## Declaration of interest

The authors declare that there is no conflict of interest that could be perceived as prejudicing the impartiality of the work reported.

## Funding

This work did not receive any specific grant from any funding agency in the public, commercial, or not-for-profit sector.

## Author contribution statement

HK was responsible for the conception and design. RO, TT, YO, MK, and TS provided administrative support. HK and RO were responsible for the provision of study materials or patients, contributed to the collection and assembly of data and were responsible for data analysis and interpretation. All authors helped in manuscript writing. All authors approved the final manuscript.
